# Targeting the Sphingolipid System as a Therapeutic Direction for Glioblastoma

**DOI:** 10.3390/cancers12010111

**Published:** 2020-01-01

**Authors:** Melinda N. Tea, Santosh I. Poonnoose, Stuart M. Pitson

**Affiliations:** 1 Centre for Cancer Biology, University of South Australia and SA Pathology, UniSA CRI Building, North Tce, Adelaide, SA 5001, Australia; melinda.tea@unisa.edu.au; 2 Department of Neurosurgery, Flinders Medical Centre, Adelaide, SA 5042, Australia; santoshpoonnoose@me.com; 3 Adelaide Medical School and School of Biological Sciences, University of Adelaide, SA 5001, Australia

**Keywords:** glioblastoma, sphingolipid, ceramide, sphingosine 1-phosphate

## Abstract

Glioblastoma (GBM) is the most commonly diagnosed malignant brain tumor in adults. The prognosis for patients with GBM remains poor and largely unchanged over the last 30 years, due to the limitations of existing therapies. Thus, new therapeutic approaches are desperately required. Sphingolipids are highly enriched in the brain, forming the structural components of cell membranes, and are major lipid constituents of the myelin sheaths of nerve axons, as well as playing critical roles in cell signaling. Indeed, a number of sphingolipids elicit a variety of cellular responses involved in the development and progression of GBM. Here, we discuss the role of sphingolipids in the pathobiology of GBM, and how targeting sphingolipid metabolism has emerged as a promising approach for the treatment of GBM.

## 1. Introduction

Glioblastoma multiforme (GBM) is the most common primary brain tumor in adults. They are highly aggressive tumors resulting in a median survival of less than 15 months from diagnosis, and a five year survival rate of 6.4%–14.0% [1,2,3]. As the term ‘multiforme’ indicates, GBM is a highly heterogeneous disease, and diagnosis has been traditionally based on histological features; however, the previously unknown molecular characteristics of the disease have now been incorporated into the World Health Organization classification of diffuse infiltrating gliomas, of which GBM is the most invasive [4,5,6]. Most cases of GBM (~95%) present the cerebral hemispheres as diffuse, highly infiltrative tumors, where the degree of malignancy is often based on the presence of features such as mitotic activity, atypical nuclei, microvascular proliferation and areas of necrosis [7,8,9]. GBM tumors usually present as a large, irregular mass that is heterogeneous in macroscopic appearance, with cystic and gelatinous areas and multifocal hemorrhage, and frequently display extensive necrosis [10]. In addition to these histopathological features, recent advances have allowed GBM to be classified based on gene expression and mutation analysis into different molecular subtypes [8,11,12]. These subtypes differ in their histological features, clinical characteristics, response to therapy and survival time. Previously, four molecular subtypes were described—Classical, Mesenchymal, Neural and Proneural—however, the Neural subtype has subsequently been eliminated, as it has been found to more closely resemble normal neural tissue, as well as lacking the characteristic gene abnormalities usually associated with GBM [11,13]. Despite the high degree of phenotypic and molecular heterogeneity observed in GBM, treatment of this disease differs little between patients, and has remained largely unchanged in the last decade. 

## 2. Current Treatment of GBM

**Figure 1:**
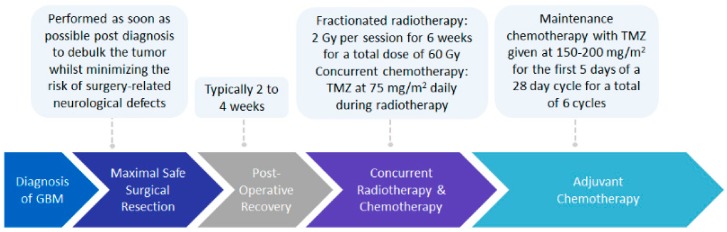
Overview of the current standard of care treatment for newly diagnosed GBM. Patients are treated according to what is commonly known as the Stupp protocol. Patients undergo the maximal safe surgical resection to remove as much of the tumor as possible, followed by a 2–4 week post-operative recovery period. Once patients have recovered from surgery, they undergo radiotherapy for a total dose of 60 Gy, with concurrent TMZ chemotherapy administered daily. Patients then receive maintenance chemotherapy with TMZ alone for a further six months. At the cessation of maintenance chemotherapy, the monitoring of patients for response to therapy and subsequent treatment options is at the discretion of the neurosurgeon.

The current standard of care treatment for GBM involves the surgical resection of the tumor, followed by post-operative radiotherapy, plus concurrent and adjuvant chemotherapy with temozolomide (TMZ), commonly known as the Stupp protocol (Figure 1) [1,2]. Maximal safe resection of the tumor, to reduce tumor mass whilst minimizing the risk of surgery-related neurological defects, has been validated to improve patient outcomes in terms of progression-free survival and overall survival [14]. Post-operative radiation involves fractionated radiotherapy of 2 Gy per session over a period of six weeks, for a total dose of 60 Gy, with a 2–3 cm margin from the gross tumor volume [1,2,15,16]. This margin is required as recurrence of almost 90% of GBM cases has been shown to occur within a 2 cm margin of the primary tumor site [1,16,17]. Oral administration of TMZ, a DNA-alkylating agent able to cross the blood–brain barrier (BBB), occurs daily during radiation therapy at a dose of 75 mg/m^2^, followed by adjuvant chemotherapy at a dose of 150–200 mg/m^2^ for the first five days of a four week cycle, for a total of six cycles [1,2,18,19]. 

The response to TMZ varies from patient to patient, largely due to the epigenetic regulation of the gene encoding the DNA repair enzyme O^6^-methylguanine-DNA methyltransferase (*MGMT*). Methylation of the *MGMT* promoter has been reported to occur in 45% of patients, resulting in the silencing of the DNA repair gene, and is a strong predictive indicator of response to TMZ and radiotherapy [2,20,21]. Interestingly, whilst some studies have shown that hypomethylation of the *MGMT* promoter does not necessarily correlate with the expression of MGMT protein levels [22,23], others have shown a correlation between *MGMT* promoter methylation and MGMT expression to be a strong predictive indicator of favorable outcomes for patients treated with TMZ [22,24,25]. The uncertainty surrounding the prognostic value of MGMT methylation status may be a consequence of the diagnostic method by which the methylation status of the promoter is determined, as well as the heterogeneity of the tumor itself, and the variability in the methylation patterns of the 98 CpG sites located within the *MGMT* promoter [26,27]. Regardless of MGMT expression/promoter methylation status, patients are usually still treated with TMZ, as limited treatment options are available that are able to traverse the BBB [8]. Despite aggressive treatment, and irrespective of initial TMZ responses, most patients succumb to the disease due to recurrence of the primary tumor through intrinsic or acquired mechanisms of resistance. This highlights the need for more effective targeted therapies [28,29].

## 3. Advances in Treatments for GBM

No major advances in the up-front treatment of GBM have been made in the last decade, and the prognosis for patients remains poor [30]. A range of therapies will likely be required to address the different aspects of GBM, such as those associated with differences between the three molecular subtypes, *MGMT* promoter methylation status, and aberrations in the signaling pathways involved in the pathogenesis of the disease. The repurposing of existing drugs, and the development of novel drugs and technology for use in combination with the current Stupp protocol, may form the basis of potential new strategies in the treatment of GBM [2]. 

The use of other chemotherapies for GBM is limited due to the common inability of these drugs to cross the BBB. To overcome this, biodegradable wafers of the alkylating agent carmustine (BCNU/1,3-bis (2-chloroethyl)-1-nitroso-urea), known commercially as Gliadel, have been placed in the resection cavity of patients at the time of surgery to slowly release the chemotherapeutic agent [30]. Typically, there is a three week period between debulking surgery and the commencement of radiotherapy, therefore the use of Gliadel wafers during this time offers the advantage of treatment during what is normally a non-therapeutic period [30]. Overall, these wafers have been shown to be relatively safe and effective in patients with primary and recurrent GBM, increasing survival time by 2–4 months [31,32]. However, there are also well documented side effects associated with the use of these wafers in the resection cavity, including defects in wound healing, cerebrospinal fluid leakage, intracranial hypertension and neurological deficits [33,34]. Consequently, as neither Gliadel or TMZ are curative, other treatment options are also under investigation.

GBM are highly vascularized tumors that express high levels of vascular endothelial growth factor (VEGF), making them an attractive target for anti-VEGF therapy [35,36]. Bevacizumab (Avastin), an anti-VEGF antibody, has been shown to be effective in the treatment of a number of solid cancers, including metastatic colorectal cancer, renal cell cancer and non-small cell lung cancer [37,38,39,40]. Bevacizumab has been approved for use in the treatment of GBM, despite reports of limited improvement in overall survival [41,42,43]. A Phase III study has reported an increase in progression-free survival and improved quality of life, however, an increase in adverse events in patients receiving bevacizumab compared to a placebo was also observed [41,42]. 

Platelet-derived growth factor (PDGF) is another pro-angiogenic factor that is considered to be a driver of GBM growth, progression and the de-differentiation of glial cells into stem cells [44,45,46]. Dasatinib is a small molecule tyrosine kinase inhibitor that, amongst other targets, inhibits PDGF receptor kinase and Src family kinases, both of which have been linked to aberrant signaling pathways in GBM [47,48]. Dasatinib has been trialed in patients with recurrent GBM, however, no benefit in survival time was observed in patients who had previously been treated with bevacizumab [49]. In a murine model of spontaneous GBM, limited efficacy of systemic administration of dasatinib has been reported, due to the active efflux of the drug via the BBB [50].

Epidermal growth factor receptor (EGFR) regulates the proliferation, growth and survival of cells through the binding of its ligand’s epidermal growth factor (EGF) or transforming growth factor-α (TGF-α) to activate signaling [51]. *EGFR* is frequently mutated in GBM and is often associated with the amplification of the *EGFR* gene, which is present in 97% of patients with the Classical molecular subtype of GBM [11,12]. The EGFR inhibitor gefitinib selectively inhibited GBM tumor cell migration in an ex vivo organotypic slice culture system of EGFR-amplified GBM tissue, however, it has shown only a minor benefit in clinical trials [52,53,54]. Whilst some improvement in overall survival was observed in these trials, this could not be correlated with EGFR expression, amplification or mutation [54,55]. Clinical trials using another EGFR inhibitor erlotinib reported limited efficacy in GBM, which appeared to be largely due to poor penetration into the brain, as a result of drug efflux by the ATP-binding cassette transporters P-glycoprotein (P-gp/MDR1/ABCB1) and Breast Cancer Resistance Protein (BCRP/ABCG2) [56]. 

Tumor-treating fields (TTFields) represent a new non-pharmacological dimension to existing modalities of cancer treatment. TTFields act through the local delivery of low intensity, alternating electric fields directly to the scalp to selectively disrupt cell division in rapidly dividing cells [57,58,59,60]. A randomized clinical trial of patients who had undergone maximal safe debulking surgery and radio- and chemotherapy, who were then trialed on maintenance therapy with either a combination of TMZ and TTFields or TMZ alone, showed that patients who received both therapies had a statistically significant increase in median overall survival time from 16.0 months to 20.9 months [30,61]. TTFields have now been approved by the US Food and Drug Administration (FDA) for use in patients with newly diagnosed and recurrent GBM, as they have been proven to be safe and effective [62]. For recurrent GBM, progression-free survival or overall survival was not extended, however, monotherapy with TTFields was associated with an improved patient quality of life, as patients did not undergo any chemotherapy and therefore did not experience chemotherapy-associated toxicity [63]. 

A number of new immunotherapeutic approaches have been recently developed and examined in GBM. These include immune checkpoint inhibitors, chimeric antigen receptor (CAR) T-cells, and dendritic cell/peptide vaccines (reviewed in [64,65,66,67]). While these approaches show considerable promise, they all currently remain in clinical trials, with the immunosuppressive tumor microenvironment of GBM representing a major barrier. Oncolytic viruses directed towards GBM have also been explored, and show therapeutic potential due to their ability to both lyse tumor cells and recruit immune responses against virally infected cells, effectively turning the immunologically ‘cold’ GBM tumors ‘hot’ [64,68]. While demonstrating remarkable efficacy in subsets of GBM patients, this approach currently remains in early-phase clinical trials [69,70].

## 4. Sphingolipid Biosynthesis and Metabolism

**Figure 2:**
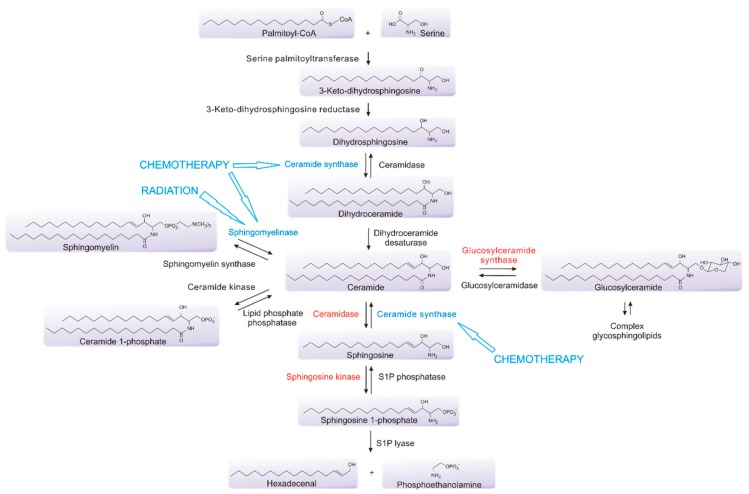
Sphingolipids act as critical bioactive signaling molecules to determine many aspects of cell fate and function. Central to the sphingolipid pathway is ceramide, a key mediator of the apoptotic response of cells to radiation and chemotherapeutic drugs. Ionizing radiation activates acid SMase to induce the conversion of sphingomyelin to ceramide. Chemotherapies including TMZ promote ceramide accumulation through multiple mechanisms, including the activation of ceramide synthases and N-SMase (shown in blue). Enhanced activity of other key enzymes involved in sphingolipid metabolism, including acid ceramidase, sphingosine kinases or glucosylceramide synthase (shown in red), represent mechanisms which GBM cells exploit to overcome existing therapies through reducing therapy-induced ceramide accumulation.

Sphingolipids are integral structural components of cell membranes that also act as critical bioactive signaling molecules to determine many aspects of cell fate and function. At their simplest, sphingolipids are composed of a long chain of sphingoid bases linked to fatty acids [71]. They are synthesized de novo from serine and palmitoyl-CoA through the action of serine palmitoyltransferase (SPT), and the subsequent involvement of a range of other enzymes, to form ceramide, which is often considered to be central to the pathway, as it connects the metabolism of multiple sphingolipids [72,73,74] (Figure 2). Ceramide can be utilized to generate sphingomyelin, an abundant and essential component of myelin which is found extensively in the central nervous system (CNS), which can be used to re-form ceramide via the action of sphingomyelinases (SMases) [75]. Ceramide can also be utilized to form complex glycosphingolipids that are also common in the CNS, or can be processed by ceramidases to generate sphingosine, which, in turn, can be phosphorylated by one of two sphingosine kinases, sphingosine kinase 1 (SK1) or sphingosine kinase 2 (SK2), to form sphingosine 1-phosphate (S1P) [72]. This phosphorylation can be reversed through the action of S1P phosphatases, or, alternatively, S1P may be metabolized by S1P lyase as the only exit point in the pathway [76,77]. Sphingolipids, particularly glycosphingolipids, are highly enriched in the brain, forming the structural components of cell membranes, and are major lipid constituents of the myelin sheaths of nerve axons, as well playing critical roles in cell signaling [74,78,79,80,81]. Glycosphingolipids are known to play a role in the differentiation of embryonic stem cells and neural stem cells, through a series of metabolic processes, resulting in the expression of sialic acid-containing glycosphingolipids, known collectively as gangliosides, in the neuronal membranes [82,83,84]. Additionally, gangliosides including GD2 and GD3 have been shown to be highly expressed on GBM stem cells and their aberrant expression is involved in driving tumor growth and survival [84,85,86]. Alterations in ganglioside expression have also been established to occur in response to the TGF-β-mediated epithelial–mesenchymal transition (EMT) in normal epithelial and cancer cell lines [87]. The pharmacological modulation of sphingolipid metabolism through the inhibition of specific enzymes within the pathway also results in EMT in mouse mammary and human bladder epithelial cell lines, as well as in human mammary carcinoma cell lines [88]. In A549 lung cancer cells, tumor necrosis factor-α or TGF-β−mediated EMT results in extensive changes in the sphingolipid metabolism, promoting a shift towards sphingolipids associated with cancer-promoting functions [89].

Sphingolipids form dynamic interactions with cholesterol to produce lipid microdomains within the plasma membrane, which in turn facilitate signal transduction in response to internal and external stimuli [90,91]. Sphingolipids including ceramide, sphingosine and sphingosine 1-phosphate (S1P) are also direct signaling molecules, regulating a variety of cellular and pathological processes [92,93,94,95]. Ceramide, and in particular C16-ceramide (one of the many forms of ceramide, usually varying in the length and saturation of the N-linked acyl chain), is generally considered to elicit pro-apoptotic or cell senescence effects through modulating the activity of a range of targets, including protein phosphatase 2A (PP2A) [96], Cathepsin D [97], stress-activated protein kinase JNK [98], and p53 [99]. Ceramides have also been shown to promote apoptosis by facilitating pore formation in the mitochondrial outer membrane to release cytochrome c [100,101,102]. Sphingosine has similar pro-apoptotic effects, at least in part through regulating the function of the 14-3-3 pro-survival adaptor proteins [103,104]. In contrast, S1P has opposing roles in enhancing cell survival and proliferation, as well as other roles in stimulating cell migration, angiogenesis and inflammation [105,106,107]. These effects of S1P, which are mediated through five S1P-selective G-protein coupled receptors, named S1P_1–5_ [92,93,94,95,108,109,110], as well as via intracellular targets such as peroxisome proliferator-activated receptor (PPAR)γ [111], telomerase [112], histone deacetylases 1 and 2 [113] and atypical protein kinase C [114]. The cellular balance and localization of these sphingolipids contributes to the determination of cell fate; therefore, disruption of this ’sphingolipid rheostat’ may lead to disease development, progression and chemotherapeutic resistance of malignancies including GBM [94,105,115,116].

## 5. Targeting Sphingolipids in GBM

The sphingolipid pathway plays a key role in the determination of cell fate, making it an attractive drug target in processes such as inflammation, cardiovascular disease, diabetes and cancer [116,117,118,119,120]. Notably, the current therapy for GBM induces multiple effects on the sphingolipid pathway. Ionizing radiation causes single and double strand breaks in DNA, but also activates acid SMase to induce conversion of the sphingomyelin found in cell membranes to ceramide [121,122,123,124]. This enrichment of ceramide in the plasma membrane results in the clustering of cell death receptors, promoting apoptosis [125,126,127,128]. 

**Figure 3:**
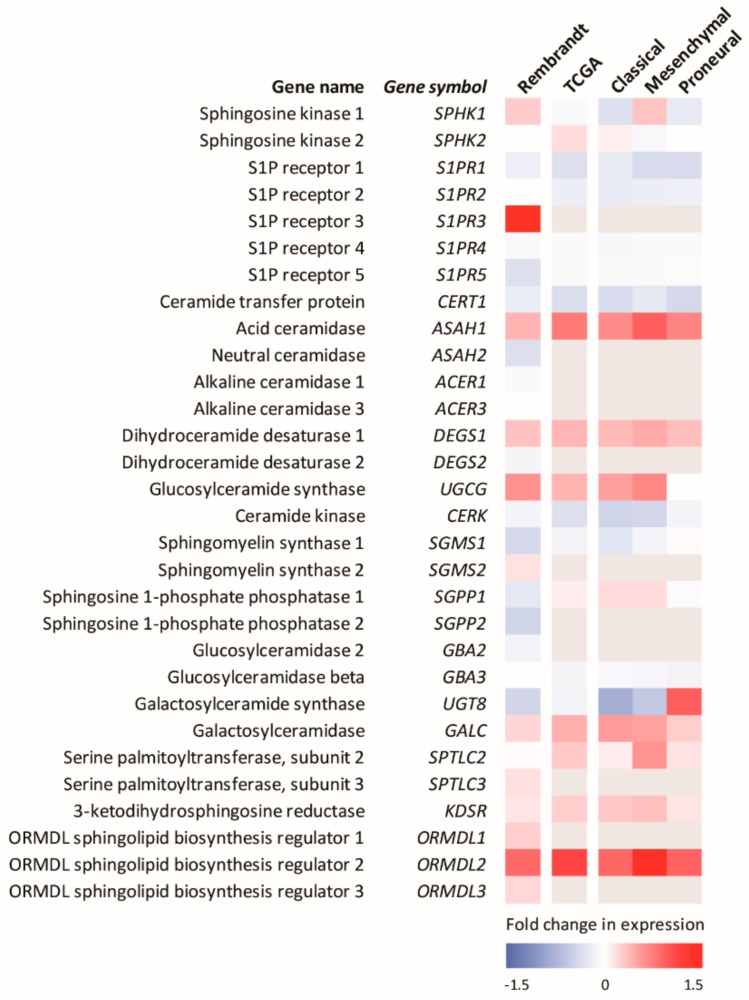
Analysis of mRNA levels of sphingolipid metabolic enzymes in GBM patients from publicly available Rembrandt and Cancer Genome Atlas brain cancer datasets show small but significant differences in gene expression compared to the normal brain. Rembrandt dataset (n = 214). TCGA dataset (n = 169), which can be subdivided into Classical (n = 54), Mesenchymal (n = 58) and Proneural (n = 57) molecular subtypes. White boxes indicate genes which are not significantly differentially expressed (*p* > 0.05). Grey boxes indicate genes for which complete data were not available. Other genes involved in sphingolipid metabolism for which no data were available were: Ceramide synthases 1-6 (*CERS1-6*), Alkaline ceramidase 2 (*ACER2*), Acid sphingomyelinase (*SPMD1*), Neutral sphingomyelinases 1 and 2 (*SPMD2* and *SPMD3*), Lipid phosphate phosphatases 1-3 (*PLPP1-3*), and Serine palmitoyltransferase, subunit 1 (*SPTLC1*).

TMZ also causes DNA double-strand breaks and has been shown to promote the accumulation of ceramide in GBM cells [125]. This is consistent with the known effects of many chemotherapies, which often activate ceramide formation through multiple mechanisms, including the activation of ceramide synthases, with this ceramide accumulation playing a major role in the mechanism of action of many of these agents [90,129,130,131,132,133,134,135]. Thus, the current therapies for GBM work in part by altering sphingolipid metabolism to enhance pro-apoptotic ceramide levels. Heightened ceramide metabolism via, for example, the enhanced levels of sphingosine kinases, acid ceramidase or glucosylceramide synthase, commonly observed in many cancers, may clear this elevated ceramide and overcome radio/chemotherapy-induced cell death, providing a mechanism for cancer resistance to these therapies [129,130,131,136]. Notably, analysis of the expression levels of sphingolipid metabolic enzymes in GBM show small but significant differences compared to the normal brain (Figure 3).

### 5.1. Ceramide-Induced Cell Death in GBM

Ceramide is a key mediator of the apoptotic response of cells to chemotherapeutic drugs. Ceramide accumulation in response to cytotoxic doses of TMZ have been observed in TMZ-sensitive GBM cell lines, while this increase in ceramide was not observed in TMZ-resistant cells [137]. Interestingly, lower levels of ceramide have been reported in tissues resected from patients with high-grade GBM relative to glial brain tumors of lower grade or normal brain tissue surrounding the tumor [138]. Patients with low ceramide levels showed an increased malignant progression of disease and poorer overall outcomes, suggesting ceramide levels are inversely correlated with patient survival and may contribute to resistance to therapy. The precise reasons behind these altered levels of tumor ceramide remain unknown, although these lipids can be generated through a variety of mechanisms that have been shown to be affected in GBM.

### 5.2. Ceramide Formation

#### 5.2.1. Dihydroceramide Desaturase 1

Dihydroceramide desaturase 1 (DES1) is responsible for the conversion of dihydroceramide (dhCer) to ceramide. The inhibition of DES1 by agents such as fenretinide results in a reduction in ceramide formation via the de novo synthetic pathway, as well as dhCer accumulation, which has been widely linked to the induction of autophagy [139,140,141,142,143,144]. As autophagy may have both pro-survival and pro-apoptotic effects, care must be taken when utilizing DES1 inhibition to modulate the balance of sphingolipids [145]. Using a range of compounds with DES1 inhibitory properties, Casasampere and colleagues showed increased dhCer levels in U87 and T98G GBM cells, correlated with the induction of autophagy [144]. In the case of U87 cells, this promoted cell survival [144]. It must be noted, however, that the compounds used to target DES1 in this study were low potency DES1 inhibitors with many other known targets, including acid ceramidase [146], making unambiguous interpretation of the findings difficult. Indeed, other studies have demonstrated that the accumulation of dihydroceramide plays a key role in mediating cytotoxic autophagy in U87 GBM cells, possibly by altering autophagosome and autolysosome permeability [147]. Consistent with this, co-treatment of a panel of GBM cells, including primary cell lines with TMZ and SKI-II, a dual inhibitor of SK1 and SK2 and also a potent DES1 inhibitor, promoted autophagy and subsequent caspase-3-dependent cell death through the accumulation of dihydrosphingosine (dhSph) and dhCer [142]. 

#### 5.2.2. Sphingomyelinases

Sphingomyelin is a major component of plasma membranes which can be hydrolyzed by acid sphingomyelinase (A-SMase) or neutral sphingomyelinase (N-SMase) to form ceramide [148,149]. These SMases can be activated by radiation and chemotherapy, respectively, to mediate ceramide-induced apoptosis [128,150,151]. Indeed, mice deficient in A-SMase are resistant to radiation- and chemotherapy-induced apoptosis [152,153].

The role A-SMase plays in response to radiation and chemotherapy in GBM is unclear. Over-expression of A-SMase in LNT-229 and T98G GBM cells did not sensitize these cells to radiation or chemotherapy with TMZ, despite causing increased ceramide accumulation [154]. Conversely, in U87 GBM cells where p53 activity was inactivated by way of HPV-16 E6 oncoprotein expression, sensitization to radiation occurred via A-SMase-induced ceramide formation [149]. Furthermore, targeting A-SMase using the small molecule inhibitor SR33557 in U87 cells with non-functional p53 activity resulted in reduced ceramide formation and the suppression of radiation-induced apoptosis [149]. A survival analysis of patients in the Glioma Rembrandt database showed no correlation between increased A-SMase mRNA levels and survival, however analysis was limited by a small number of patients. Further analysis of the larger TCGA GBM dataset showed low A-SMase levels correlated with increased survival [154]. 

SMases also exert apoptotic effects in GBM cells via the generation of reactive oxygen species (ROS). Over-expression of A-SMase has been reported to sensitize U373MG cells to the chemotherapy gemcitabine via ceramide-dependent ROS production [155]. Furthermore, treatment of U87, U373 and T98G GBM cells lines with etoposide caused ROS formation, N-SMase activation and ceramide-induced apoptosis [156]. This effect was negated when p53 was mutated via site-directed mutagenesis, and no significant changes in ceramide levels were observed in response to treatment with etoposide [156]. Interestingly, the opposite effect was observed in response to γ-radiation [157]. U87 GBM cells with functional p53 were found to be resistant to γ-radiation-induced apoptosis, but in the background of mutant p53, caspase-3-induced apoptosis was triggered by increased ceramide production via A-SMase, rather than N-SMase, activation [157].

### 5.3. Ceramide Metabolism

#### 5.3.1. Glucosylceramide Synthase

Glucosylceramide synthase (GCS) is involved in the glycosylation of ceramide to form glucosylceramide, a precursor of complex glycosphingolipids [158,159]. Cell stress responses, such as cell cycle arrest and apoptosis, can be induced in response to elevated cellular ceramides, and, as such, these responses may be lost as ceramides are converted to glycosphingolipids, promoting cell survival and drug resistance [159,160]. This is supported by evidence from mouse glioma cell lines where, unlike in gemcitabine sensitive cells, resistance to gemcitabine results in a failure to accumulate ceramide in response to chemotherapy [161]. Instead, the ceramide in these cells appears to be rapidly metabolized through GCS, and inhibition of this enzyme using small molecule inhibitors or siRNA-mediated gene silencing is able to reverse these effects, resulting in ceramide accumulation and cell death [161]. 

Increases in GCS mRNA and glucosylceramide levels have also observed in T98G cells engineered to be resistant to TMZ and Paclitaxel [137]. Co-treatment of these chemo-resistant GBM cells with small molecule GCS inhibitors D-*threo*-1-phenyl-2-decanoylamino-3-morpholino-1-propanol (PDMP), and DL-*threo*-1-phenyl-2-palmitoylamino-3-morpholino-1-propanol (PPMP) or N-(n-nonyl)deoxygalactonojirimycin (N-DGJ) with either TMZ or Paclitaxel, increased cell death, suggesting GCS inhibition sensitizes cells to chemotherapy [137]. A similar effect was reported in parental T98G and LNT-229 GBM cells, where the inhibition of GCS by PPMP promoted cytotoxicity and reduced clonogenicity [154]. Furthermore, treatment of parental T98G and LNT-229 cell lines or LNT-229 cells cultured to become TMZ-resistant with a combination of PPMP and TMZ, or PPMP and irradiation, resulted in additive, but not synergistic, effects on cell viability [154]. Mechanistically, GSC has been reported to confer chemotherapy resistance of GBM cells through the suppression of chemotherapy-induced ceramide accumulation, which in turn attenuates the NADPH oxidase-dependent generation of ROS that is required to induce the cytotoxic effects of chemotherapy [162]. 

#### 5.3.2. Acid Ceramidase

Acid ceramidase (ASAH1), is the rate-limiting enzyme involved in the conversion of ceramide to sphingosine, and plays an important role in regulating the amount of sphingosine available for conversion to S1P [158]. The elevated transcription of *ASAH1* has been reported in a number of cancers, including GBM, and has been found to confer resistance to apoptosis [93,163,164]. 

Increased ASAH1 expression in response to γ-radiation has been reported in U87 cells with non-functional p53 activity [149]. Subsequent inhibition of acid ceramidase using *N*-oleoylethanolamine, was found to suppress this increase in ASAH1 expression, resulting in increased ceramide accumulation [149]. Elevated ASAH1 levels have been reported in the tumors of patients with newly diagnosed GBM, as well as radiotherapy-treated GBM, correlating with poor survival [165,166]. This elevation of ASAH1 in response to radiation has also been reported in both adult (U87) and pediatric (SJGBM-2) GBM cell lines, as well as patient tissue samples [165,166]. The mechanism by which this resistance occurs is thought to be through a reduction in ceramide accumulation, and an upregulation of S1P, resulting in increased cell viability [166]. Notably, inhibition of either ASAH1 or S1P using neutralizing antibodies reduced cell viability in the U87 and SJGBM-2 cells, which had previously been exposed to radiation [166]. 

ASAH1 levels are also elevated in the subpopulation of GBM cells known as glioblastoma stem-like cells, which express the neural and brain cancer stem cell marker CD133, which in turn is associated with poor prognosis if expressed at high levels [165,166,167,168,169,170,171]. Glioblastoma stem-like cells are also thought to contribute to recurrence of disease, given their self-renewal and tumor-initiating abilities [167,172,173,174]. CD133-positive glioblastoma stem-like cells are thought to confer resistance to radiation through preferential upregulation of DNA damage checkpoint kinases, which more effectively repair the DNA damage induced by radiation [175]. 

Inhibition of ASAH1 using the drug carmofur, a pyrimidine analogue and derivative of fluorouracil, has been used in the treatment of colorectal cancer, resulting in increased ceramide and apoptosis [176,177]. A similar response has been reported in radio-sensitive and radio-resistant GBM cells lines, where increases in intracellular ceramide in response to carmofur resulted in cell death [166]. Carmofur has shown some ability to permeabilize the brain, however, the extent to which this may occur in patients with brain tumors is unknown [176,178]. Despite this, the inhibition of ASAH1 is a promising new target in the treatment of GBM and may represent a novel marker of radiotherapy resistant GBM [179]. 

#### 5.3.3. Ceramide Synthase

Ceramide synthases comprise a family of six enzymes (CerS1-6) which selectively generate ceramides with different N-linked acyl chain lengths [116,180,181]. CerS1 preferentially generates C18-ceramide, CerS2 generates C22-, C24- and C26-ceramide, CerS3 generates very long (C26-) to ultra-long (>C32-) ceramides, CerS4 generates C18- and C20-ceramides, whereas CerS5 and CerS6 generate C14- and C16-ceramide [181,182]. While once considered equivalent, advances in the last two decades have shown that different ceramide species have distinct cellular roles [181,183]. For example, long chain (e.g., C16- and C18-) ceramides are commonly associated with the induction of apoptosis, while very-long chain (e.g., C24-) ceramides have anti-apoptotic properties [183,184]. The ceramide synthases display heterogenous tissue distribution and have been established to play a role in cancer and chemoresistance [181,185]. 

CerS1 is the most highly expressed CerS in the central nervous system [183], where it generates C18-ceramide [186]. Levels of C18-ceramide have been shown to be reduced in glioma patient samples compared to normal brain tissue [186], and, in GBM specifically, C18-ceramide is reduced by 70% compared to non-tumor tissue [187]. Supplementing U251 or A172 GBM cell lines with exogenous C18-ceramide, or the overexpression of CerS1, was shown to cause cell death via the induction of endoplasmic reticulum (ER) stress, leading to lethal autophagy, and through inhibition of the PI3K/AKT signaling pathway, and also sensitized these cells to the chemotherapeutic Teniposide [186].

The production of ceramide by ceramide synthases at the mitochondrial membrane plays a critical role in apoptosis, as ceramide contributes to the insertion of the Bcl2-associated X protein (Bax) into mitochondrial membranes, resulting in mitochondrial outer-membrane permeabilization (MOMP) and, ultimately, cell death [101]. An atypical member of the Bcl-2 family, known as Bcl2L13, is elevated in a number of cancers including GBM, and over-expression of this protein in an orthotopic xenograft mouse model of GBM is associated with disease progression and decreased survival [188]. In vitro, Bcl2L13 was shown to exert its effects through inhibition of the pro-apoptotic enzymes CerS2 and CerS6, upstream of Bax activation and MOMP [188]. Knockdown of Bcl2L13-expression in the orthotopic xenograft model resulted in prolonged survival. 

CerS6 also plays a role in GBM cell death induced by interleukin (IL)-24, a cytokine known to have anti-tumor functions [189,190,191]. IL-24 exerts its cytotoxic effects through the induction of ER stress, resulting in an increased ceramide formation in primary GBM cell lines [192]. In addition, IL-24 sensitized GBM cells to ionizing radiation [185], and showed synergy with histone deacetylase inhibitors to induce lethal autophagy in primary GBM cells in a CerS6-dependent manner [193]. Taken together, these data suggest that the modulation of ceramide and ceramide synthases may be a viable therapeutic target in GBM

### 5.4. Sphingosine 1-Phosphate-Mediated Cell Signaling

S1P has roles in cell survival, migration and angiogenesis, and is known to play an important role in cancer [194,195,196,197,198]. 

#### 5.4.1. Sphingosine 1-Phosphate and S1P Receptors

There is extensive evidence supporting the critical roles in which S1P-mediated signaling contributes to the pathogenesis of GBM [199]. S1P has been established to promote the migration and invasion of GBM cells in vitro via S1P signaling through G-protein coupled S1P receptors expressed on GBM cells [93,200,201,202]. S1P also plays a key role in angiogenesis [93,194]. Extracellular S1P derived from glioblastoma stem-like cells in vitro has also been found to confer resistance to TMZ, independent of MGMT status, emphasizing the importance of S1P levels in GBM [203]. Sphingolipidomics has shown S1P to be 9-fold higher, and ceramide 5-fold lower, in GBM patient samples compared to normal grey matter, and elevated S1P has been correlated with poor patient survival [93,200]. 

S1P receptors S1P_1_, S1P_2_, S1P_3_ and S1P_5_ are expressed by GBM cells, whereas expression of S1P_4_ has not been detected, consistent with the restricted expression of this receptor, mainly in the lymphoid compartment [200,204,205,206,207]. There is conflicting evidence as to the role of S1P receptors in GBM. In T98G and G112 GBM cell lines which express high levels of S1P_1_, siRNA knockdown of S1P_1_ promoted S1P-mediated cell proliferation [205]. Consistent with this, over-expression of S1P_1_ in U251 and U87 GBM cell lines, which express low levels of this receptor, reduced cell growth in vitro and reduced tumor growth in an orthotopic model, with no changes in migration or invasion observed in these cells [205]. S1P_1_ and S1P_3_ are, however, known to promote GBM cell migration and S1P-mediated cell invasion [200,208,209]. S1P_2_, however, appears to have a negative effect on GBM cell migration, although these findings did not extend to invasiveness, as the overexpression of S1P_2_ in U-118 MG GBM cells in vitro was able to induce the expression of proteins which interact with extracellular matrix components to promote GBM cell invasion [209].

The relationship between S1P receptor levels and patient survival is also unclear. *S1P_1_*, *S1P_2_*, *S1P_3_* and *S1P_5_* mRNA levels have been shown to be increased in GBM patients [200,204]. Increased *S1P_1_* mRNA has been positively associated with prolonged GBM patient survival [200]. Other studies, however, have found decreased *S1P_1_* mRNA and protein levels in GBM tissue compared to the normal brain. Although consistent with the findings above, this downregulation of *S1P_1_* was associated with poor GBM patient survival [205]. Increased *S1P_2_* mRNA levels have been shown to be negatively associated with GBM patient survival, whereas increased *S1P_3_* and *S1P_5_* had no observable effect [200]. However, a different study showed that low *S1P_5_* mRNA levels are positively associated with increased survival of GBM patients [204]. In vitro overexpression of S1P_5_ has been shown to inhibit GBM proliferation [209]. 

#### 5.4.2. Therapeutic Modulation of Sphingosine 1-Phosphate Signaling 

The therapeutic drug FTY720, also known as fingolimod, is a structural analogue of sphingosine and is currently used to treat forms of relapsing multiple sclerosis due to its immunosuppressive effects, mediated via its engagement of S1P receptors [210,211,212]. In this context, FTY720 is a pro-drug, being phosphorylated in vivo by sphingosine kinase 2 to generate FTY720-P, which is the form of the drug that engages four of the S1P receptors (S1P_1,3–5_). FTY720-P initially acts as an agonist of these receptors, but subsequently leads to their prolonged internalization and degradation, and thus works as a functional antagonist of S1P receptors. The action of FTY720-P on S1P_1_ induces lymphopenia by blocking the egress of lymphocytes from secondary lymphoid organs [213]. FTY720/FTY720-P, however, can target multiple components of sphingolipid metabolism, leading to the dysregulation of ceramide, sphingosine and S1P [214,215,216,217]. In particular, FTY720 has been shown to inhibit all six ceramide synthases, as well as sphingosine kinase 1, and S1P lyase [214]. 

FTY720 has been proposed as a therapeutic option for the treatment of GBM, as it is able to cross the blood–brain barrier (BBB) due to its lipophilic nature, and is able to accumulate in brain tissue and cerebrospinal fluid [212,218,219]. In vitro, FTY720 has been shown to induce apoptosis in multiple GBM cell lines, as well as inhibit migration and invasion through the modulation of matrix metalloproteinases [220,221,222]. FTY720 has been shown to sensitize U251 and U87 GBM cells to TMZ via suppression of the redox-sensitive transcription factor nuclear factor erythroid 2-related (Nrf2) [223], which is known to induce autophagy and apoptosis in U251 cells in response to TMZ [224,225]. Nrf2 promotes U251 cell migration and invasion in vitro and is required to maintain the self-renewal capacity of glioblastoma stem-like cells [226,227]. The involvement of S1P receptors in these effects of FTY720, however, remains unclear. 

Treatment of mice with FTY720 in a flank xenograft model of GBM showed reduced tumor growth through induction of autophagy, apoptosis and necroptosis in the tumor cells [221]. These studies were subsequently extended into orthotopic models of GBM using brain tumor stem cells derived from patient samples, where FTY720 treatment resulted in reduced tumor growth and prolonged mouse survival [228]. Furthermore, in these models, mice treated with both FTY720 and TMZ showed increased survival, above that of mice treated with either therapy alone. 

FTY720 has been tested for safety in conjunction with radiotherapy and TMZ in a Phase 0 clinical trial for newly diagnosed GBM (ClinicalTrials.gov Identifier: NCT02490930). One aim of the trial was to reduce the lymphopenic effects of standard radiotherapy and chemotherapy, which can persist for up to 12 months post treatment [229]. It was proposed that administration of FTY720 prior to the initiation of standard radiotherapy and chemotherapy may reduce this therapy-induced lymphopenia by temporarily sequestering leukocytes in the secondary lymphoid organs during radio/chemotherapy. However, despite the trial having been completed, the investigators have yet to report their findings. Other means of therapeutic targeting of S1P receptors remain to be investigated.

#### 5.4.3. Sphingosine Kinases

Targeting sphingosine kinases (SKs) with SK inhibitors in combination with radiotherapy and/or chemotherapy has been widely proposed as a potential approach for cancer therapy [230,231,232,233]. SK-mediated conversion of the pro-apoptotic ceramide induced by radiotherapy and chemotherapy into S1P has been implicated in GBM resistance to therapy [93,234]. The use of SK inhibitors following radiotherapy or chemotherapy-induced ceramide production has been proposed as a means to reduce this SK-mediated conversion of ceramide to S1P [233]. 

SK1 is upregulated in GBM and its expression correlates with poor patient survival [93,200,235]. The use of highly selective SK1 inhibitors have shown inconsistent effects in reducing GBM cell proliferation and viability in vitro [93,236]. However, in an orthotopic mouse model of GBM, mice treated with the SK1-specific inhibitor SK1-I showed reduced tumor growth and vascularization, resulting in increased mouse survival [236].

The SK inhibitor SKI-II (also known as SKi) has been shown to induce apoptosis in GBM cells, including TMZ-resistant cells, and to suppress GBM cell migration and invasion [236,237]. Furthermore, when used in combination with TMZ and radiation, SKI-II showed strong cytotoxic effects against U87 GBM cells in vitro [238]. However, while SKI-II was initially reported to be a SK1 specific inhibitor, it has since been found to also inhibit SK2 and be a potent DES1 inhibitor [239,240,241]. Thus, the true mechanisms responsible for the effects of SKI-II remain undefined. 

SK2 is highly expressed in the brain and produces the majority of S1P found in the brain [242]. However, the expression of SK2 is not consistently upregulated in patients with GBM, and its role in cancer is generally less well understood [93,200,204,243]. The efficacy of SK2-selective inhibitors in GBM has not been widely studied, however, siRNA-mediated knockdown of SK2 has been shown to reduce GBM cell proliferation and survival to a greater extent than SK1 knockdown [235]. The subcellular localization of SK2 has been shown to be important for the function of SK2, particularly in cancer [244]. Recent studies have shown SK2 localization to be regulated by the cytoplasmic protein cytoplasmic dynein 1 intermediate chain 1 (DYNC1I1), which is highly downregulated in GBM, with low DYNC1I1 associated with poor patient prognosis [245]. This loss of DYNC1I1 in GBM enhances SK2 localization to the plasma membrane [245], a localization shown to enhance oncogenic signaling by this enzyme [244]. Consistent with this, re-expression of DYNC1I1 in the tumor cells reduced the plasma membrane localization of SK2, and reduced U251 GBM tumor growth in mice. Furthermore, the treatment of mice with the selective SK2 inhibitor K145 reduced the subcutaneous tumor growth of U251 GBM cells, providing further evidence that targeting sphingolipid metabolism in GBM may be a viable therapeutic option [245].

## 6. Conclusions

The lack of major advances in the treatment of GBM to date has meant that the prognosis for patients remains poor. Current challenges in finding effective therapies for GBM include the high degree of tumor heterogeneity, the presence of glioblastoma-like stem cells, and difficulties in drug delivery due to the BBB and drug efflux, all of which have been extensively reviewed by others [246,247,248,249]. Given the complexity of GBM, and the challenges associated with treatment, it is likely that combination therapies will be required to improve outcomes for patients. Advancements in the identification of prognostic markers, further extensive molecular characterization of tumors and improved preclinical models of GBM provide a promising outlook for improving the prognosis for patients with GBM. Given the well established and emerging roles that sphingolipids play in the biology of GBM, targeting the sphingolipid metabolism represents a viable approach for the treatment of GBM.
